# Comparative Efficacy of Simulation-Based and Traditional Training in Ultrasound-Assisted Regional Anesthesia for Medical Students: Randomized Controlled Trial

**DOI:** 10.2196/77702

**Published:** 2026-02-03

**Authors:** David Sánchez-Poveda, Juan Vicente-Mampel, Belén Curto, Vidal Moreno, Juan A García-Esteban, Felipe Hernández-Zaballos, Pablo Alonso-Hernández

**Affiliations:** 1 Pain Unit Anaesthesiology Service Complejo Asistencial Universitario de Salamanca (CAUSA) Salamanca Spain; 2 Department of Physiotherapy School of Medicine and Health Science Valencia Catholic University Saint Vincent Martyr Valencia Spain; 3 Department of Computer Science and Automation University of Salamanca University of Salamanca Salamanca Spain

**Keywords:** education, ultrasound, anesthesia, nerve block, simulation

## Abstract

**Background:**

Ultrasound is very important in medicine and teaching, but there are not many formal training programs. We also do not know much about what students think. To be good at using ultrasound, one needs to learn technical, thinking, and seeing skills. This is especially true in regional anesthesia (RA), where mistakes in reading images can cause problems. Training with simulations is a safe and good way to learn these skills. Some models are helpful for teaching how to perform procedures using ultrasound.

**Objective:**

This study aimed to evaluate the effectiveness, localization time, and success rate of traditional teaching versus a new simulation-based teaching method for RA designed by the investigators among undergraduate medical students.

**Methods:**

A prospective, randomized controlled trial was conducted at the University of Salamanca from April 2022 to January 2023. A total of 34 medical students in their fourth to sixth academic years were randomly allocated to either a simulation-based training group using the Haptic Ultrasound Probe or a traditional teaching group. The simulation approach used a realistic probe replica and a software-based ultrasound environment, whereas the traditional method comprised a theoretical lecture and curated audiovisual materials. Two days after training, participants underwent a blinded assessment requiring the identification of peripheral nerve plexuses using an ultrasound device. The primary outcome measured was the successful identification of nerves, and the secondary outcome was the time taken to complete each procedure. Data were analyzed using an intention-to-treat approach.

**Results:**

A total of 34 medical students (fourth to sixth years) were recruited to compare traditional teaching with simulation-based training in ultrasound-guided nerve localization. No statistically significant differences were found in the success rates between the groups. For the interscalene approach, the traditional teaching group achieved a 100% (17/17) success rate compared to 82% (14/17) in the simulation group (*P*=.07). The time to task completion was similar across most procedures. In the sciatic nerve division, the traditional teaching group was significantly faster, with a mean time of 42.4 (SD 39.5) seconds (*P*=.02). The regression models showed no significant interaction between the intervention type and academic year. Both teaching methods had positive educational impacts.

**Conclusions:**

Simulation-based learning effectively supports competency acquisition in RA and offers a safe, scalable alternative to traditional methods. Its integration into medical curricula may standardize training, improve skill consistency, and enhance patient safety. Further multicenter studies with larger, diverse cohorts are needed to validate these benefits and guide implementation in medical education.

## Introduction

Medical specialists are increasingly using ultrasound technology for diagnostic purposes and to guide therapeutic procedures owing to its numerous clinical advantages [1]. Some researchers have characterized portable ultrasound as the “visual stethoscope” of the 21st century [2], highlighting its growing relevance in modern medicine. Point-of-care ultrasound is a portable and versatile imaging modality that allows clinicians to perform rapid bedside assessments in various clinical scenarios. It is accessible and efficient [3,4]. Point-of-care ultrasound has become an essential tool in emergency medicine, critical care, and other specialties [5,6]. Consequently, ultrasound training has been incorporated into the undergraduate medical curriculum, encompassing both theoretical instruction and practical hands-on experience. However, the American Institute of Ultrasound in Medicine reports that only approximately one-third of medical schools in the United States have implemented specialized ultrasound training programs. In addition, there is a noticeable gap in the literature regarding medical students’ views on optimal practices for ultrasound education [7]. Furthermore, there is limited understanding of whether its use enhances the acquisition of medical knowledge independent of technical skill development [8].

To effectively acquire ultrasound competencies, medical students must master a combination of technical, cognitive, and perceptual skills. Key areas include proper probe handling, such as the ability to maneuver the probe across anatomical surfaces, and the development of a 3D orientation to accurately visualize and interpret internal structures. Additionally, students must integrate real-time imaging with their anatomical knowledge to make precise adjustments during the examination, which is critical for both diagnostic accuracy and therapeutic efficacy [9]. Equally important is visual training, which enables students to recognize the anatomical structures within ultrasound images. This process requires repeated exposure and active problem-solving to enhance spatial awareness and the ability to discern subtle differences between tissues and organs [10]. These visual and interpretive skills must be grounded in a strong understanding of anatomy to ensure accurate interpretations.

Motor coordination also plays a pivotal role, particularly in interventional procedures that involve inserting needles into targeted soft tissues, where precision is essential to avoid complications. In the field of regional anesthesia (RA), the use of ultrasound has increased significantly over the past few decades because of its ability to enhance the safety and accuracy of procedures that were traditionally guided by anatomical landmarks and neurostimulation [11]. However, to fully realize these safety benefits, trainees must receive comprehensive education on the use of ultrasound technology as the equipment alone cannot guarantee a safe outcome. The number and complexity of RA techniques have also expanded, with some procedures requiring advanced sonoanatomical knowledge for successful performance [9]. In response to this evolution, Regional Anaesthesia UK (the UK division of the European Society of Regional Anaesthesia and Pain Therapy) proposed a classification system that groups RA techniques according to the level of knowledge and expertise required for their performance. Notably, most malpractice incidents associated with RA result from misinterpretation of ultrasound images or inadequate anatomical exploration [12].

Simulation-based learning provides valuable opportunities to develop these competencies in controlled settings. It allows both undergraduate and postgraduate students to practice without the ethical and logistical challenges of performing procedures on live patients or cadavers while still preserving the tactile feedback necessary for motor skill development [13]. Simulation training also enables repetition, feedback, and safe failure, which are key components of effective procedural training. As such, it has emerged as a highly promising approach for training in ultrasound-guided procedures. Several simulation-based teaching models have demonstrated their effectiveness in developing RA skills [14,15]. In this context, our research group developed a novel ultrasound simulator, the Haptic Ultrasound Probe (HUSP), which was officially registered on April 23, 2018, with the Spanish Trademark and Patent Office (*Oficina Española de Marcas y Patentes*) [16]. This study aimed to evaluate the effectiveness of a simulation-based teaching method using HUSP compared to traditional instruction for teaching RA to undergraduate medical students [1].

## Methods

### Study Design

This study was designed as a prospective, longitudinal, randomized controlled trial and conducted in accordance with the CONSORT (Consolidated Standards of Reporting Trials) guidelines (Multimedia Appendix 1) [17]. Participants were randomly and equally assigned to either a simulation-based education group or a traditional educational method group. Data were collected between April 2022 and January 2023.

### Ethical Considerations

Participation was voluntary, and all students provided informed consent before their inclusion in this study. The study adhered to the ethical principles of the Declaration of Helsinki [18] and was approved by the institutional review board of the Department of Surgery, Faculty of Medicine and Health Sciences, University of Salamanca (protocol code 001-2018; July 13, 2018). Measures were implemented to protect participant privacy and confidentiality, including the anonymization of all collected data and secure storage of records. No compensation or incentives were provided to students.

### Sample Size Calculation

This study used convenience sampling by recruiting participants who were easily accessible in a university. While this approach allows for efficient data gathering, it is crucial to recognize that it may limit the generalizability of the study due to potential selection biases. Therefore, an intention-to-treat analysis was performed to address this concern.

### Participants

A total of 34 students from the Faculty of Medicine at the University of Salamanca (medical degree program) participated in this study. A sample was selected including those from the fourth, fifth, and sixth years of study. Both data collectors and analysts were unaware of the assigned interventions. The sample consisted of 32% (n=11) male and 68% (n=23) female individuals distributed in 2 independent groups. The inclusion criteria were as follows: (1) students enrolled in the medical degree program at the University of Salamanca; (2) students in the fourth, fifth, or sixth year; (3) students who voluntarily provided informed consent; (4) students willing to participate in all activities and assessments of the study; and (5) students with sufficient cognitive and physical capabilities to participate in the teaching interventions (simulation based).

### Randomization and Blinding

All educational sessions were conducted by 2 instructors with extensive experience (>10 years) in ultrasound-guided RA following standardized teaching protocols. One of the researchers delivered both the traditional and simulation-based interventions, whereas the second researcher acted as an independent evaluator and remained blinded to group allocation. To minimize bias, group assignment was randomized using a table generated by an independent researcher in Microsoft Excel. A double-blind methodology was implemented; neither the participants nor the evaluator had knowledge of the group allocation or performance outcomes until the end of the study.

### Procedures

This study investigated the simulation of interscalene, supraclavicular, and sciatic nerve blocks, including the external and internal popliteal divisions of the nerves, as they appear as Plan A and Plan B blocks in the Regional Anaesthesia UK guidelines. An Esaote MyLab Alpha ultrasound device, identical to that used to generate simulation ecosystem images, was used in the experiment. Two individuals with plexuses that were confirmed to have adequate ultrasound visibility before testing took part in the study. All participants were scheduled for an assessment 2 days after their simulation training with the HUSP simulator. The assessment required participants to identify plexuses in 3 live individuals. To ensure impartiality in student group allocations, a blind investigator who was not involved in providing the theoretical lesson or guiding the simulation ecosystem practice administered the tests. The time taken by each student to locate the plexus was recorded, and successful identification was noted (maximum identification time: 120 seconds). Any attempt exceeding 120 seconds was considered unsuccessful in locating the nerve plexus.

### Educational Material

#### Traditional Educational Method

After a theoretical presentation by an expert, the students were exposed to instructor-curated audiovisual content to augment their comprehension. These materials included videos addressing the 3 principal topics covered in the theoretical lectures. The videos were carefully selected to complement the theoretical content by providing practical demonstrations and visual explanations. The instructor had the option of using YouTube videos, which are often freely accessible and exhibit a range of real-world examples, or consulting a textbook on RA or nerve blocks, which typically offers a more structured and academic perspective with detailed and evidence-based information. The choice between these resources depended on the teaching methodology and the desired depth of the material. While YouTube videos can present practical, real-life applications and demonstrations, textbooks generally provide a more comprehensive and authoritative source of knowledge supported by scholarly references.

#### Simulation Educational Method

Following a concise theoretical introduction by the same expert, the simulation group engaged in a 2-hour practical session at the Faculty of Science, University of Salamanca. During this session, the participants practiced identifying the brachial plexus using the simulation system.

The ultrasound imaging system used comprised theoretical and practical components.

**Figure 1:**
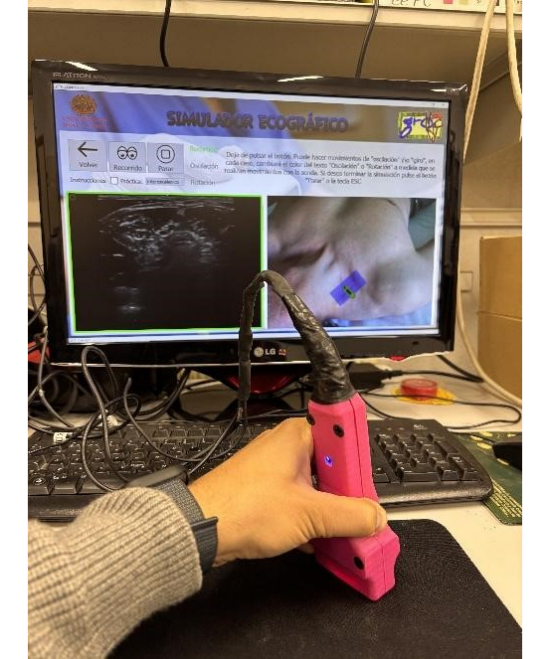
Illustration of the probe's movements as recorded by the software, which are used to enhance the precision of the ultrasound-guided approach.

The Ultrasound Desktop Educational Simulator helped students in acquiring ultrasound images, comprehending the spatial relationship between anatomical structures in the ultrasound image and the overall 3D view of the patient’s anatomy, and coordinating hand movements with structure visualization. A significant feature of the Ultrasound Desktop Educational Simulator was a realistic ultrasound probe replica that trainees could operate as if it were an actual ultrasound transducer (Figure 1).

**Figure 2:**
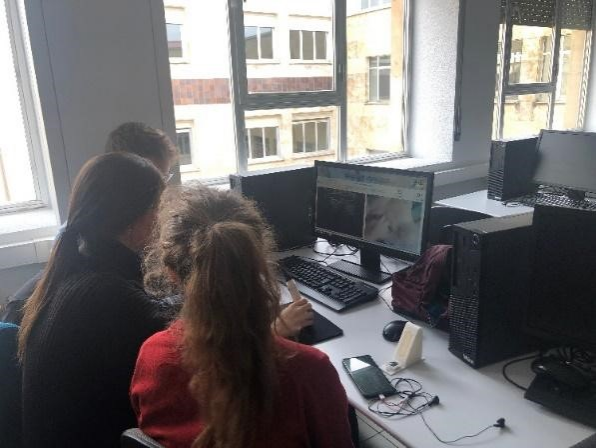
Simulated work environment designed for students, allowing them to perform tasks collaboratively in small groups.

The simulation system screen displayed a 2D image of the corresponding anatomical pathways. The movement of the probe induced changes in both the skin surface and ultrasound images (Figure 2).

**Figure 3:**
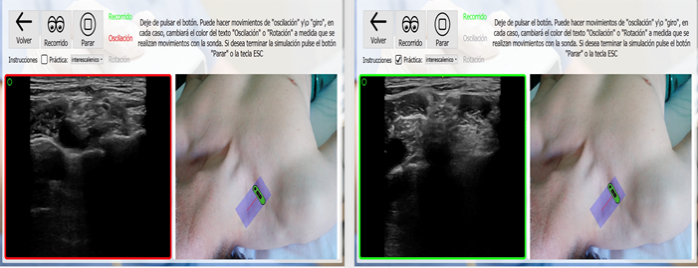
Depiction of the student highlighted in green, indicating correct probe positioning for the intervention.

A guide informed the students when they captured correct images (Figure 3). The simulation tool used in this study comprised 2 components: a probe replica that emulated a real ultrasound transducer and a multiprogram desktop application. The probe replica, designated as HUSP by its developers, contained sensors that monitored the trainee’s hand movements. The software application generated ultrasound images that were prerecorded using real equipment based on the trainee’s probe manipulations. It also enabled students to capture ultrasound images, anatomical surface images, and probe paths. Screen borders appeared when the student obtained correct ultrasound images. On April 23, 2018, the Spanish Trademark and Patent Office granted utility model number 201700521 to protect the HUSP device [16].

### Outcome Measures

The primary outcome variable was the success rate of ultrasound-guided peripheral nerve identification, recorded as a binary outcome (success or failure). The secondary outcome was the time (in seconds) required to complete each nerve localization procedure. The procedures were analyzed across 4 anatomical approaches: interscalene, supraclavicular, popliteal, and sciatic nerve division. The academic year of the students (fourth, fifth, or sixth year) was also included.

### Statistical Analysis

All data are expressed as the mean and SD. Descriptive statistics were used to summarize the participants’ demographic characteristics (age, sex, and academic year) and procedure times (in seconds). Categorical variables were reported as absolute frequencies and percentages. Data normality was assessed by visually inspecting histograms. Additionally, the Shapiro-Wilk test was performed to formally evaluate the normality of the data. Given the pilot nature of this study and the limited number of participants per group, an intention-to-treat analysis was performed [19]. The primary outcome, success in nerve identification, was evaluated using the chi-square or Fisher exact tests to compare the proportions between the traditional and simulation-based teaching groups for each anatomical approach. For the secondary outcome (procedure time), mixed linear models were applied to assess the mean differences between groups, including the variable “academic year” as a covariate. This allowed for the evaluation of potential confounding and interaction effects on the results of the study. Interaction terms (group × time) were tested to explore whether the effectiveness of the intervention varied over time [19]. Unstandardized coefficients, 95% CIs, and *P* values were reported for all regression models. A significance level of *P*<.05 was considered statistically significant. All statistical analyses were conducted using the JASP software (version 0.16.3).

## Results

### Recruitment, Participant Flow, and Sample Characteristics

**Figure 4:**
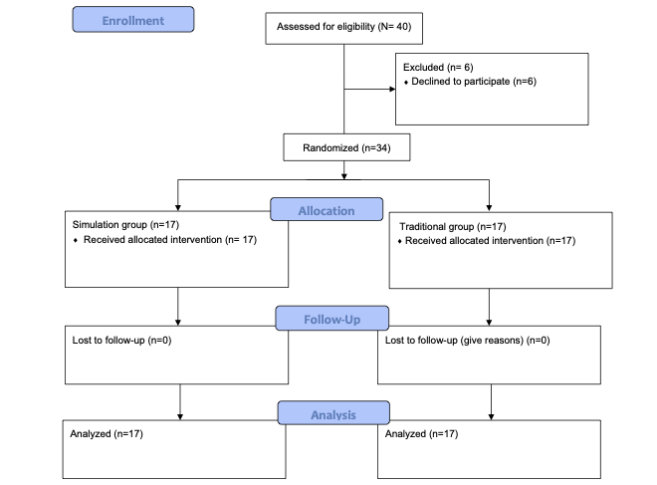
Flowchart of the participants in this study.

A cohort of 34 students from the Faculty of Medicine at the University of Salamanca (medical degree program) was recruited for this study. All students provided voluntary consent to participate (Figure 4).

The cohort comprised students in the fourth, fifth, and sixth years of the medical degree program. Comprehensive demographic data, including course year, sex, and age, are presented in Table 1. Furthermore, detailed information stratified by course year and duration of each ultrasound procedure is provided in Table 2.

**Table 1:** Demographic characteristics of undergraduate medical students participating in a randomized educational intervention study comparing a teaching simulator with traditional education for regional anesthesia training.

Variable		All participants (N=34)	Teaching simulator (n=17)	Traditional education (n=17)
**Sex, n (%)**				
	Male	11 (32)	5 (29)	6 (35)
	Female	23 (68)	12 (71)	11 (65)
Age (years), mean (SD)		21.8 (3.3)	22.41 (1.3)	22.4 (0.9)
**Year of study, n (%)**				
	Fourth	11 (32)	6 (35.3)	5 (29)
	Fifth	15 (44)	7 (41)	8 (47)
	Sixth	8 (24)	4 (23.5)	4 (24)

**Table 2:** Comparison of procedure duration across different ultrasound-guided nerve block approaches between undergraduate medical students trained using a simulator-based method and traditional education.

Approach	Educational intervention (s), mean (SD)	
	Teaching simulator	Traditional education
Interscalene	38.6 (46.2)	31.41 (28.5)
Supraclavicular	51.2 (41.2)	55.7 (41.2)
Popliteal	37.8 (36.3)	42.4 (39.5)
Sciatic nerve division	10.8 (14.5)	24.1 (36.8)

### Success Rate of the Intervention

The results revealed no significant differences in successful nerve localization across the various approaches between the traditional teaching and simulation groups. Specifically, in the interscalene approach, 100% (17/17) of the students in the traditional teaching group successfully located the brachial plexus compared to 82% (14/17) in the simulation group, where 18% (3/17) of the students did not succeed. However, no statistically significant difference was observed (*P*=.07). Similarly, in the supraclavicular approach, both groups demonstrated comparable success, with 18% (3/17) of the students in each group unable to locate the relevant structure. In popliteal sciatic nerve localization, slightly fewer students in the simulation group (1/17, 6%) failed than in the traditional teaching group (2/17, 12%); however, this difference was not statistically significant (*P*=.07). Finally, for sciatic nerve division, all students in the simulation group successfully identified the division, whereas 12% (2/17) of the students from the traditional group, one from the fourth year and one from the fifth year, were unable to do so.

### Supraclavicular Approach

**Figure 5:**
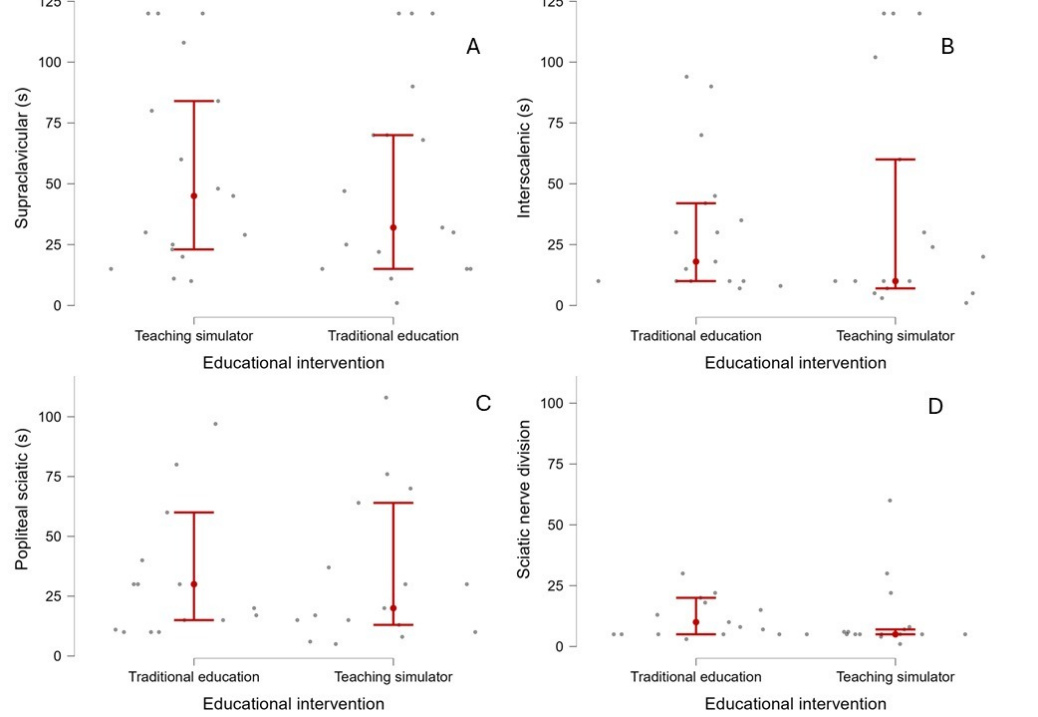
Scatterplots showing the time (in seconds) required to perform 4 types of nerve blocks according to the educational intervention: teaching simulator vs traditional education (mean and CI).

Specifically, there were no statistically significant differences based on the educational interventions received. The reported values correspond to unstandardized coefficients (β), which represent the effect of each intervention in the original units of the outcome variable along with their *P* values and 95% CIs. Both educational interventions had positive impacts: the simulator group achieved β of 106.72 (95% CI 10.06-203.37; *P*=.03), whereas the traditional teaching group achieved β of 102.80 (95% CI 5.03-200.57; *P*=.40; Figure 5A). The academic year covariate in the regression model did not interact with the effect of the educational interventions (β=−10.43, 95% CI −29.79 to 8.91; *P*=.28).

### Interscalenic Approach

The analysis revealed no statistically significant differences based on the educational methods used. Both intervention groups (traditional education and simulator-based education) exhibited positive effects. The simulator-based group achieved β of 79.38 (95% CI 24.78-164.73; *P*=.01), whereas the traditional teaching group achieved β of 74.36 (95% CI 9.03-145.23; *P*=.03; Figure 5B). In the regression model, the academic year covariate did not interact with the effect of the educational interventions (β=−8.69, 95% CI −22.39 to 4.52; *P*=.14).

### Popliteal Sciatic Nerve Approach

The analysis demonstrated no statistically significant differences based on the educational methods. The simulator-based group attained β of 98.93 (95% CI 32.54-188.50; *P*=.07), whereas the traditional teaching group achieved β of 105.46 (95% CI 27.59-195.32; *P*=.01; Figure 5C). In the regression model, the academic year covariate did not interact with the effect of the educational interventions (β=−12.63, 95% CI −28.91 to 1.33; *P*=.08).

### Sciatic Nerve Division Approach

The analysis demonstrated statistically significant differences based on the educational method used. The simulator-based group attained β of 60.72 (95% CI −3.63 to 125.07; *P*=.06), whereas the traditional teaching group achieved β of 74.67 (95% CI 9.57-139.77; *P*=.02; Figure 5D). Consequently, the traditional educational intervention demonstrated a statistically significant effect, whereas the simulator-based intervention did not reach statistical significance. In the regression model, the academic year covariate did not interact with the effect of the educational interventions (β=−10.22, 95% CI −23.11 to 2.66; *P*=.08).

## Discussion

### Principal Findings

This study evaluated the effectiveness of a simulation-based educational tool (HUSP) compared to traditional teaching for the acquisition of RA competencies in undergraduate medical students. Overall, no statistically significant differences were observed between the 2 groups regarding successful nerve localization across most approaches. In the interscalene approach, 100% (17/17) of the students in the traditional group successfully identified the brachial plexus compared with 82% (14/17) in the simulation group. Similar results were observed for the supraclavicular and popliteal approaches. In the sciatic nerve division approach, all students in the simulation group succeeded, whereas 12% (2/17) of the students in the traditional group failed, reflecting a statistically significant advantage of simulation-based training over traditional teaching in this specific case. No significant interaction was observed between the academic year and intervention type.

These findings align with those of previous studies supporting the use of simulation technologies for teaching ultrasound-guided procedures [20-22]. Simulation allows for safe and standardized practice of complex procedures and has been shown to improve knowledge retention and practical skill acquisition among medical students [23]. In this study, the HUSP simulator enabled students to reach level 2 of the Kirkpatrick evaluation model, focusing on the acquisition of knowledge and skills following educational intervention. Specifically, students learned to identify the relevant anatomical landmarks for nerve block procedures using ultrasound. Achieving higher levels of clinical competence requires complementary training involving needle use, as found in cadaver laboratories or high-fidelity simulators. During this study, an additional software module integrating needle simulation was developed, enhancing the potential of the HUSP simulator to support advanced skill acquisition.

Chen et al [2] demonstrated the effectiveness of simulation-based learning and emphasized the need for clarification of optimal simulation modalities. Other studies have reported that using live models or fresh cadaver limbs in elective courses can improve anatomical understanding and provide a valuable clinical context [24]. The HUSP simulator offers an effective alternative, especially when access to live models or cadavers is limited in the clinical setting. Its customizable design allows educators to create tailored simulation exercises, which is a pedagogical advantage. The recent integration of needle simulation hardware has further improved clinical applicability. In terms of cost-effectiveness, the simulator is affordable; the hardware is low cost and compatible with any PC, with the software license being the main investment [25]. These characteristics make it a viable educational solution, particularly in resource-constrained settings [26].

A key strength of this study is its contribution to improving ultrasound education during the preclinical stage by addressing gaps identified in previous research [27]. Despite the growing interest in integrating ultrasound into undergraduate curricula, previous studies have faced limitations such as restricted access to resources, faculty availability, and methodological rigor [28]. The development of a simulator incorporating needle simulation represents a significant step toward comprehensive interventional training for the HUSP system. This study has several limitations. First, the relatively small sample size, dictated by the fixed academic cohort, may limit the generalizability of the findings, a common challenge in similar educational studies [15]. Second, the lack of blinding and the dual role of some authors as both instructors and researchers could have introduced bias. Furthermore, an intention-to-treat analysis was performed, ensuring that all participants were analyzed in their originally assigned groups regardless of protocol adherence, thereby minimizing bias and better reflecting real-world educational conditions.

### Conclusions

Simulation-based learning is an effective tool for supporting the acquisition of competencies in RA and offers a practical, safe, and scalable alternative to traditional educational methods. Beyond individual skill acquisition, integrating simulators such as HUSP into medical curricula could standardize training, reduce variability in learner performance, and enhance patient safety by preparing students for clinical practice in a controlled environment. To strengthen the evidence and broaden its applicability, further multicenter studies with larger and more diverse cohorts are warranted, which may inform best practices and guide the implementation of simulation-based approaches in medical education programs.

## Data Availability

The datasets generated or analyzed during this study are available from the corresponding author on reasonable request.

## Appendix

Multimedia Appendix 1 CONSORT checklist.

## Abbreviations

CONSORT: Consolidated Standards of Reporting Trials
HUSP: Haptic Ultrasound Probe
RA: regional anesthesia
